# P-1272. Assessing the Impact of Penicillin Susceptibility on Biofilm Production and Identifying asa1 Expression in Clinical Enterococcus faecalis Blood Isolates

**DOI:** 10.1093/ofid/ofaf695.1462

**Published:** 2026-01-11

**Authors:** Julian Azzouzi, Lora Todorova, Olivia Funk, Thomas Inzana, Alexey Kozlenkov, Carlos Chacon, Dianjun Cao, Jaclyn A Cusumano

**Affiliations:** Arnold & Marie Schwartz College of Pharmacy and Health Sciences, Long Island University, Brooklyn, NY; Arnold & Marie Schwartz College of Pharmacy and Health Sciences, Long Island University, Brooklyn, NY; Arnold & Marie Schwartz College of Pharmacy and Health Sciences, Long Island University; James J. Peters VA Medical Center, Brooklyn, New York; Lewyt College of Veterinary Medicine, Long Island University, Brookville,, New York; James J. Peters VA Medical Center; Icahn School of Medicine at Mount Sinai, New York, New York; James J. Peters VA Medical Center, New York, New York; Lewyt College of Veterinary Medicine, Long Island University, Brookville,, New York; Long Island University, Brooklyn, New York

## Abstract

**Background:**

*Enterococcus faecalis* infective endocarditis (IE) mortality without surgical biofilm removal is up to 75%. Further complicating treatment, borderline-penicillin (PCN)-resistant, ampicillin-susceptible *E. faecalis* (borderline-PRASEF; PCN MIC 4-8 µg/mL) reduces standard-of-care ampicillin-ceftriaxone activity *in vitro*. Bacterial adhesion is the first stage of biofilm formation and is mediated by *asa1* in *E. faecalis,* but the exact relationship to biofilm formation is unknown.

**Figure:**
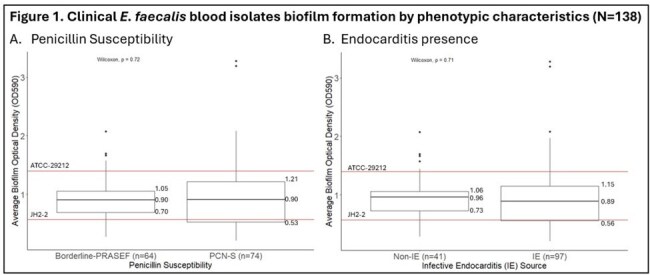

**Figure:**
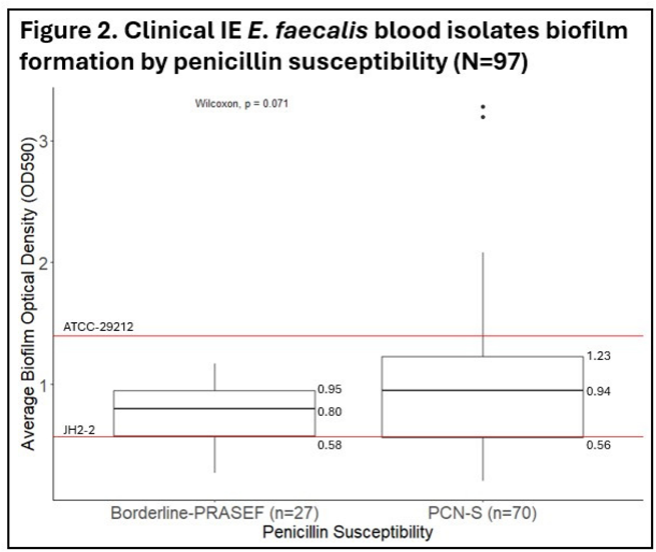

**Methods:**

Biofilm mass was quantified in 138 clinical *E. faecalis* blood isolates from New York, NY; Albany, NY; and Barcelona, Spain, including 97 from patients with IE. PCN susceptibility was assessed by broth microdilution, identifying 64 borderline-PRASEF and 74 PCN-susceptible (PCN-S, MIC ≤ 2 µg/mL) isolates. ATCC 29212 and JH2-2 served as high and low biofilm controls, respectively. Biofilms were grown for 72 hours at 35°C in 96-well plates with tryptic soy broth 1% glucose and quantified via spectrophotometry (optical density, OD_590_). Biofilm mass was compared by PCN susceptibility and IE presence using Wilcoxon rank-sum test. A subset analysis of only IE isolates compared biofilm mass by PCN susceptibility. The presence and expression of *asa1* was assessed by whole genome DNA sequencing and de novo assembly (Nanopore) and RNA sequencing (Illumina) of the three highest and three lowest biofilm formers and two controls, from planktonic culture.

**Figure:**
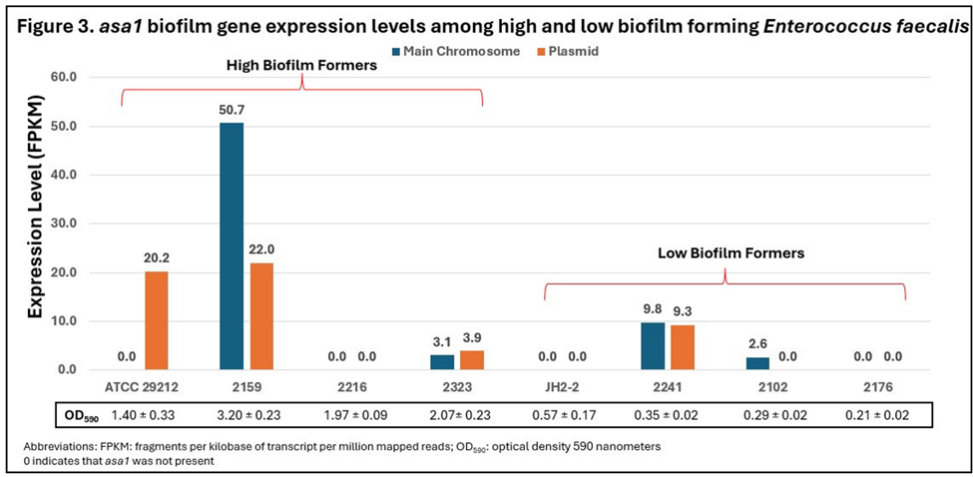

**Results:**

Median biofilm mass was similar between PCN-S and borderline-PRASEF isolates, and between IE and non-IE isolates [Figure 1A/B]. Among IE only isolates, PCN susceptibility was more common, and the median biofilm mass was higher vs. borderline-PRASEF [Figure 2]. *asa1* was expressed on either the chromosome and/or plasmid in three high and two low biofilm producers [Figure 3]. The *asa1* expression level in low biofilm formers was lower.

**Conclusion:**

*E. faecalis* PCN susceptibility and IE presence may be independent factors of biofilm formation, however borderline-PCN resistance trended towards an inverse relationship to IE presence and biofilm formation. *asa1* expression showed a strong trend towards higher *asa1* levels among high biofilm formers but further analysis of additional biofilm genes is warranted.

**Disclosures:**

Jaclyn A. Cusumano, PharmD, BCIDP, Basilea Pharmaceutica: Grant/Research Support

